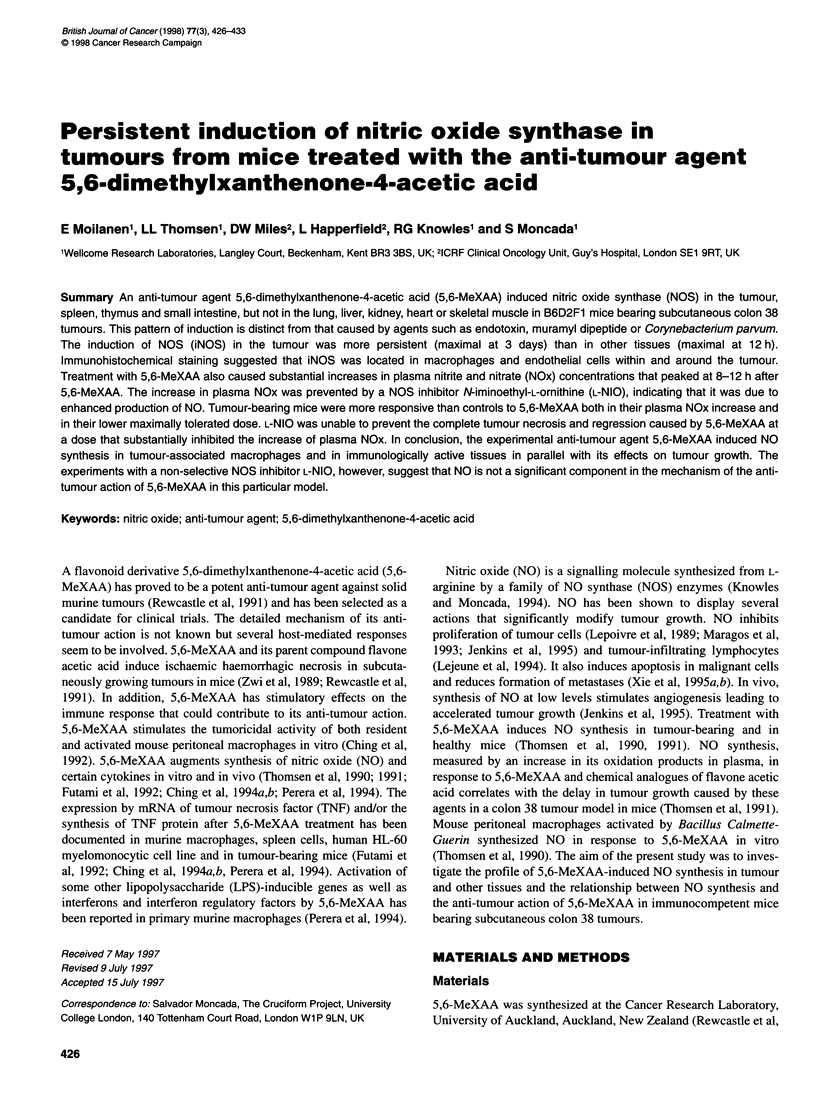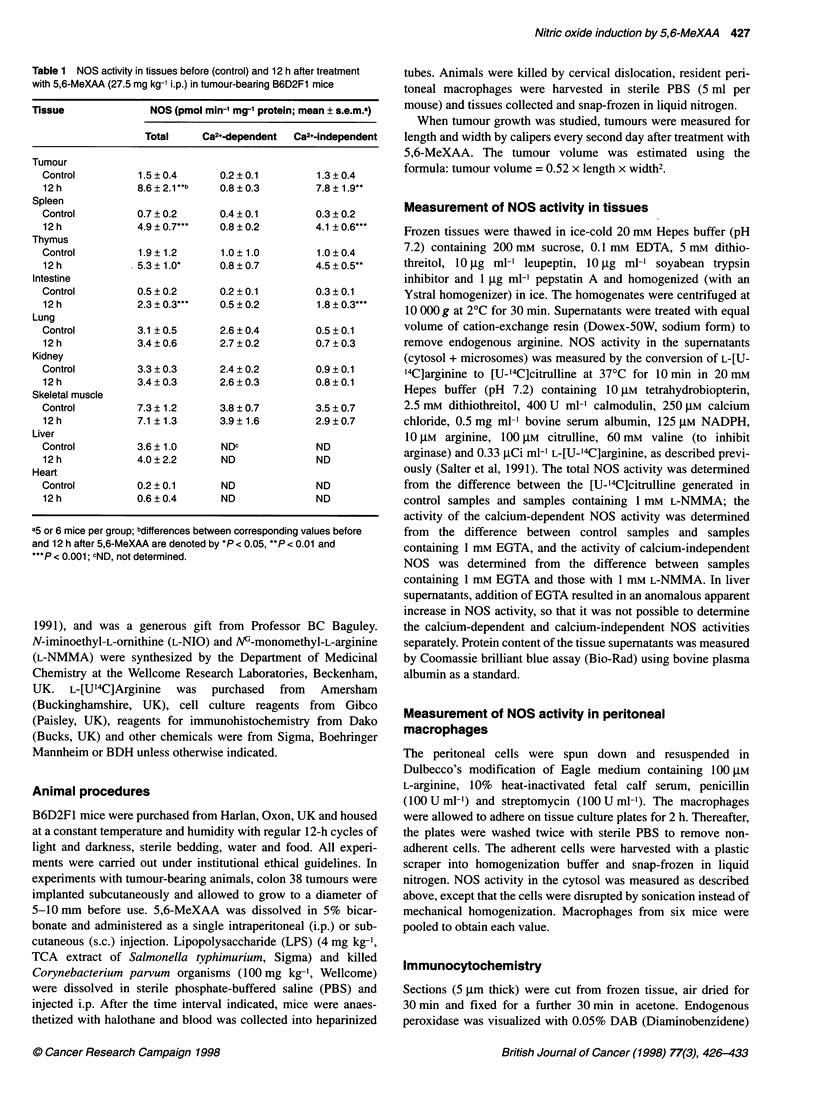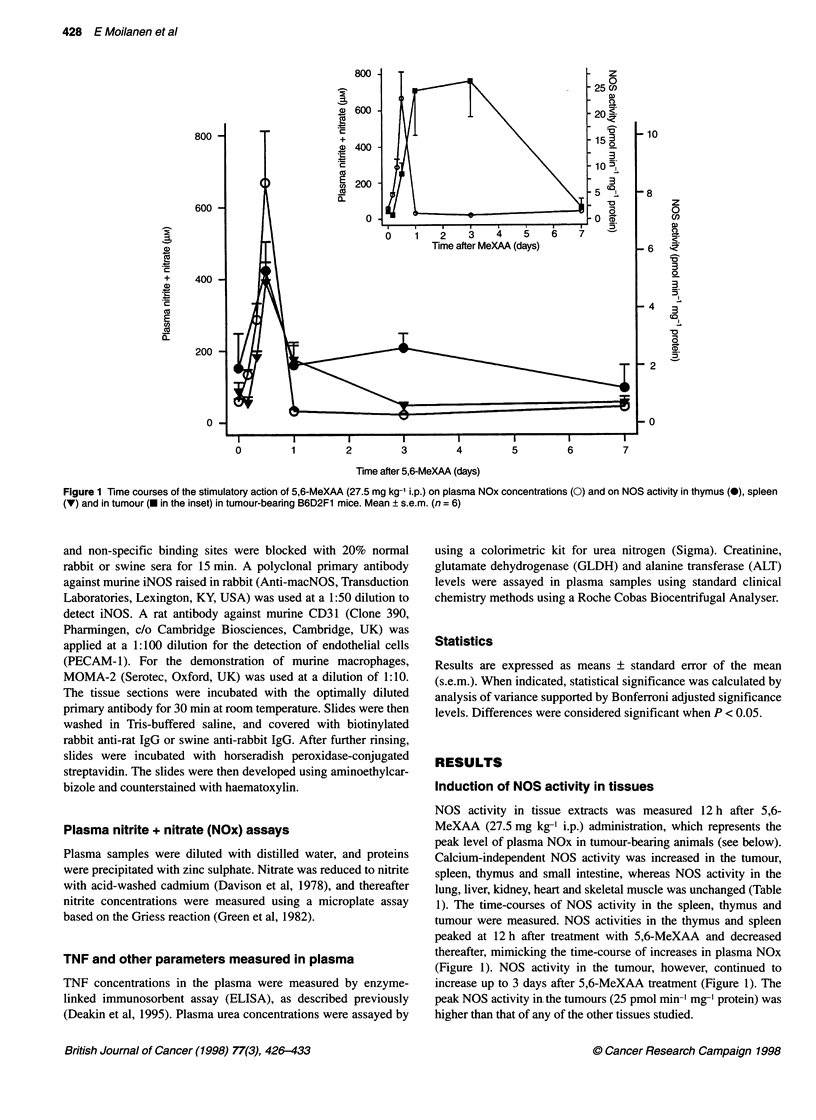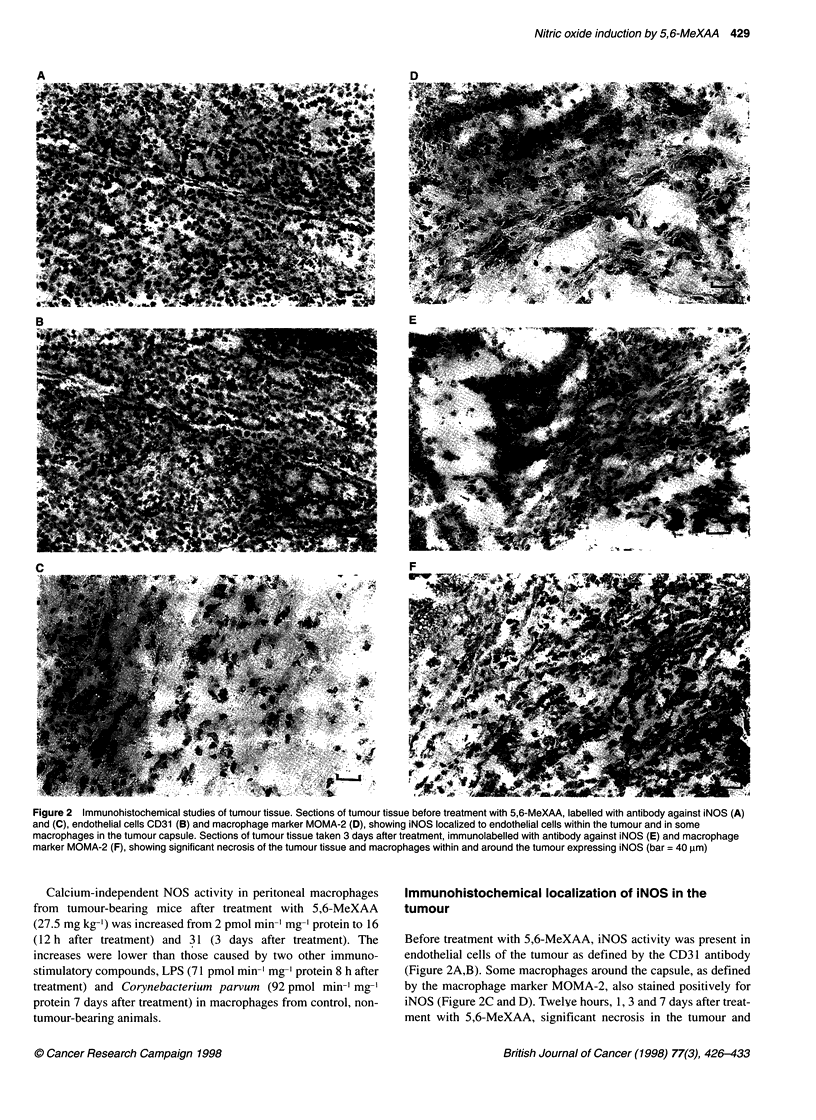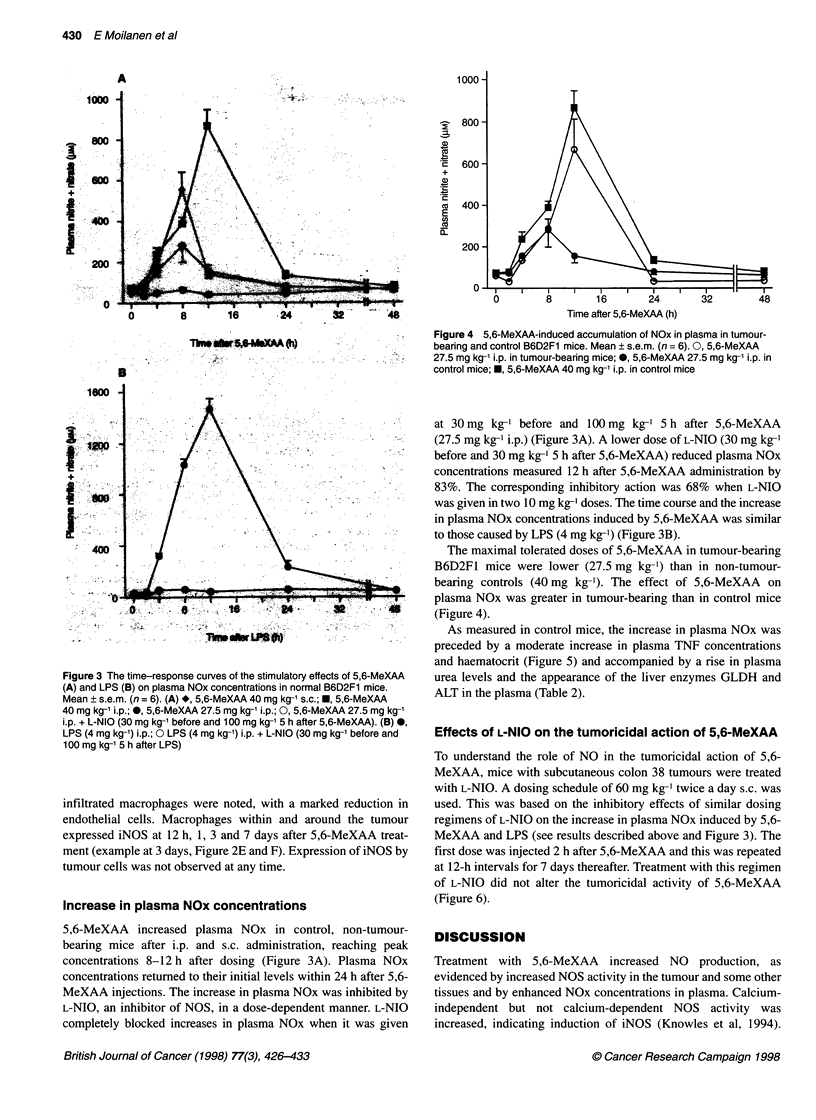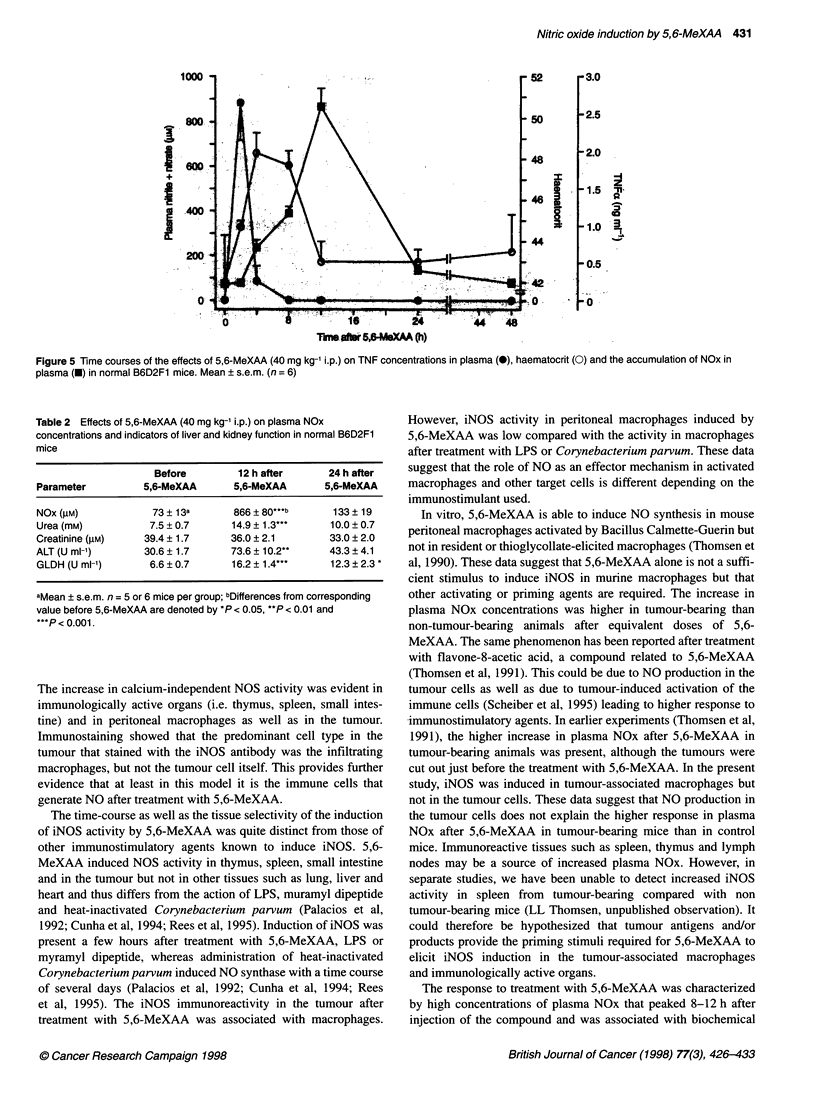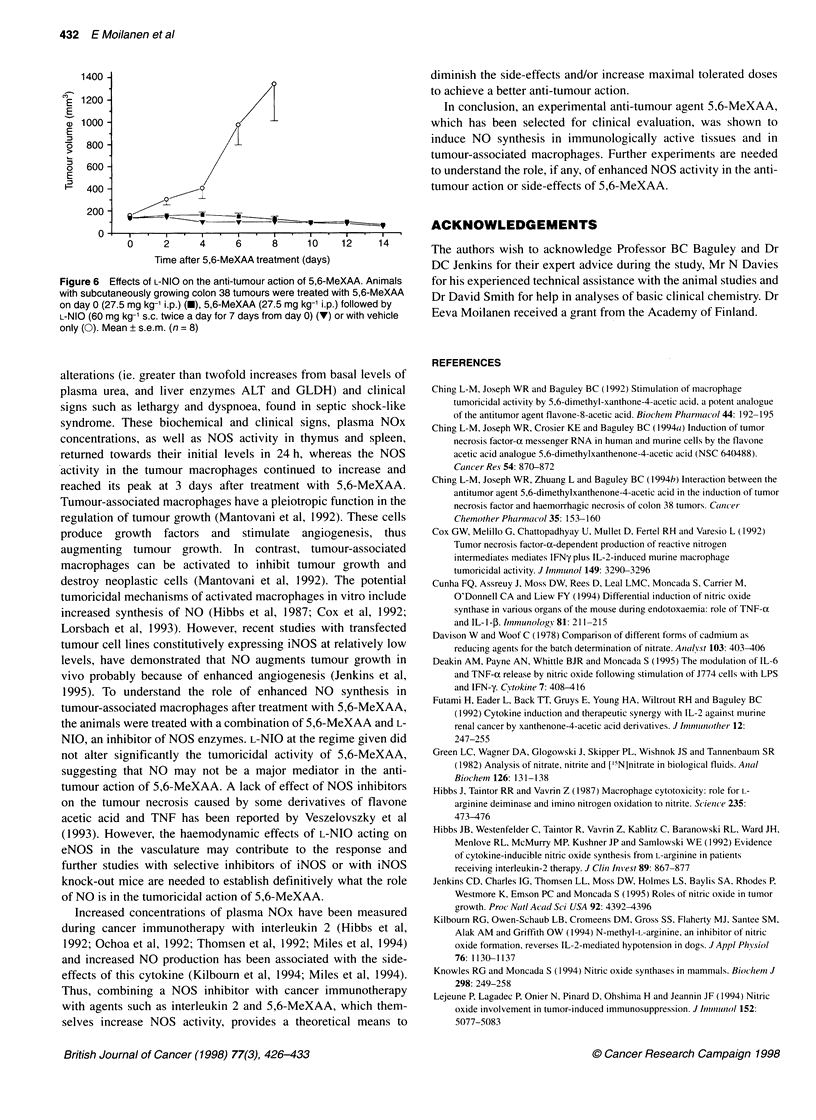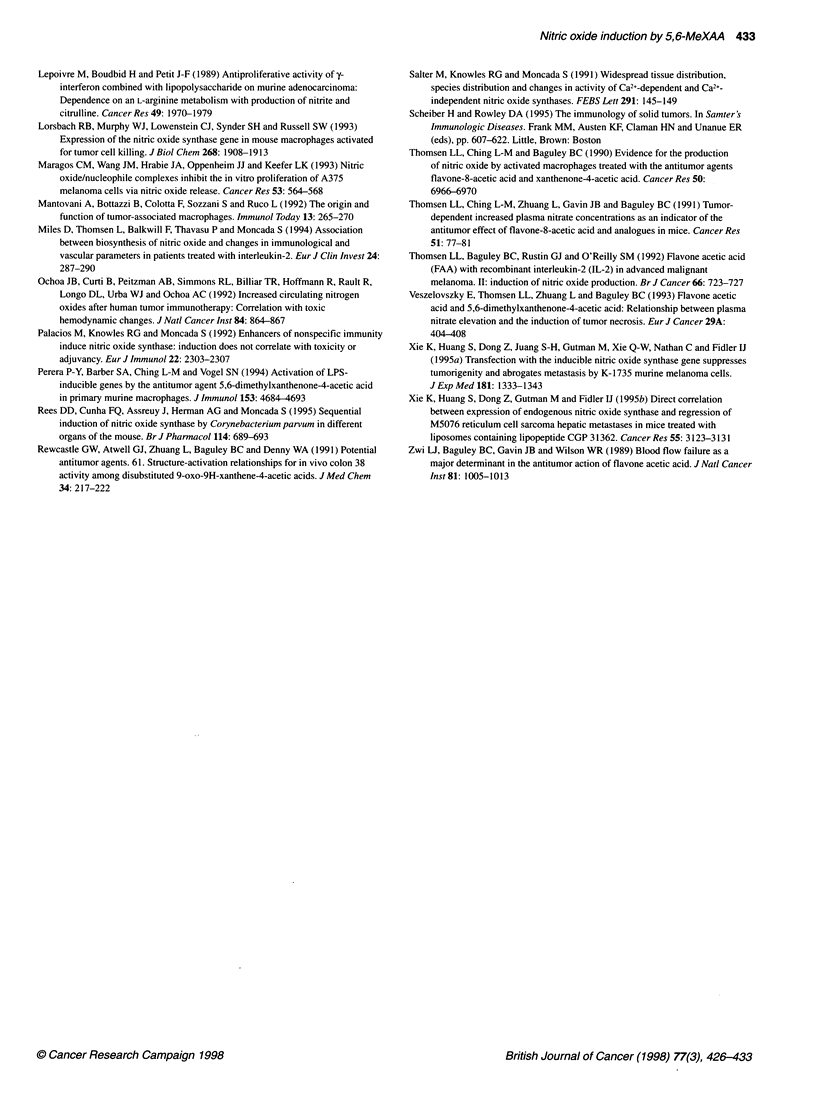# Persistent induction of nitric oxide synthase in tumours from mice treated with the anti-tumour agent 5,6-dimethylxanthenone-4-acetic acid.

**DOI:** 10.1038/bjc.1998.68

**Published:** 1998

**Authors:** E. Moilanen, L. L. Thomsen, D. W. Miles, D. W. Happerfield, R. G. Knowles, S. Moncada

**Affiliations:** Wellcome Research Laboratories, Beckenham, Kent, UK.

## Abstract

**Images:**


*British Journal of Cancer* (1998) **77**(3), 426–433
© 1998 Cancer Research Campaign

### Persistent induction of nitric oxide synthase in tumours from mice treated with the anti-tumour agent 5,6-dimethylxanthenone-4-acetic acid

**E Moilanen[1], LL Thomsen[1], DW Miles[2], L Happerfield[2], RG Knowles[1] and S Moncada[1]**

[1]Wellcome Research Laboratories, Langley Court, Beckenham, Kent BR3 3BS, UK; [2]ICRF Clinical Oncology Unit, Guy's Hospital, London SE1 9RT, UK

**Summary** An anti-tumour agent 5,6-dimethylxanthenone-4-acetic acid (5,6-MeXAA) induced nitric oxide synthase (NOS) in the tumour, spleen, thymus and small intestine, but not in the lung, liver, kidney, heart or skeletal muscle in B6D2F1 mice bearing subcutaneous colon 38 tumours. This pattern of induction is distinct from that caused by agents such as endotoxin, muramyl dipeptide or *Corynebacterium parvum*. The induction of NOS (iNOS) in the tumour was more persistent (maximal at 3 days) than in other tissues (maximal at 12 h). Immunohistochemical staining suggested that iNOS was located in macrophages and endothelial cells within and around the tumour. Treatment with 5,6-MeXAA also caused substantial increases in plasma nitrite and nitrate (NOx) concentrations that peaked at 8–12 h after 5,6-MeXAA. The increase in plasma NOx was prevented by a NOS inhibitor *N*-iminoethyl-L-ornithine (L-NIO), indicating that it was due to enhanced production of NO. Tumour-bearing mice were more responsive than controls to 5,6-MeXAA both in their plasma NOx increase and in their lower maximally tolerated dose. L-NIO was unable to prevent the complete tumour necrosis and regression caused by 5,6-MeXAA at a dose that substantially inhibited the increase of plasma NOx. In conclusion, the experimental anti-tumour agent 5,6-MeXAA induced NO synthesis in tumour-associated macrophages and in immunologically active tissues in parallel with its effects on tumour growth. The experiments with a non-selective NOS inhibitor L-NIO, however, suggest that NO is not a significant component in the mechanism of the anti-tumour action of 5,6-MeXAA in this particular model.

**Keywords:** nitric oxide; anti-tumour agent; 5,6-dimethylxanthenone-4-acetic acid

A flavonoid derivative 5,6-dimethylxanthenone-4-acetic acid (5,6-MeXAA) has proved to be a potent anti-tumour agent against solid murine tumours (Rewcastle et al, 1991) and has been selected as a candidate for clinical trials. The detailed mechanism of its anti-tumour action is not known but several host-mediated responses seem to be involved. 5,6-MeXAA and its parent compound flavone acetic acid induce ischaemic haemorrhagic necrosis in subcutaneously growing tumours in mice (Zwi et al, 1989; Rewcastle et al, 1991). In addition, 5,6-MeXAA has stimulatory effects on the immune response that could contribute to its anti-tumour action. 5,6-MeXAA stimulates the tumoricidal activity of both resident and activated mouse peritoneal macrophages in vitro (Ching et al, 1992). 5,6-MeXAA augments synthesis of nitric oxide (NO) and certain cytokines in vitro and in vivo (Thomsen et al, 1990; 1991; Futami et al, 1992; Ching et al, 1994*a,b*; Perera et al, 1994). The expression by mRNA of tumour necrosis factor (TNF) and/or the synthesis of TNF protein after 5,6-MeXAA treatment has been documented in murine macrophages, spleen cells, human HL-60 myelomonocytic cell line and in tumour-bearing mice (Futami et al, 1992; Ching et al, 1994*a,b*, Perera et al, 1994). Activation of some other lipopolysaccharide (LPS)-inducible genes as well as interferons and interferon regulatory factors by 5,6-MeXAA has been reported in primary murine macrophages (Perera et al, 1994).

*Received 7 May 1997*
*Revised 9 July 1997*
*Accepted 15 July 1997*

*Correspondence to:* Salvador Moncada, The Cruciform Project, University College London, 140 Tottenham Court Road, London W1P 9LN, UK

Nitric oxide (NO) is a signalling molecule synthesized from L-arginine by a family of NO synthase (NOS) enzymes (Knowles and Moncada, 1994). NO has been shown to display several actions that significantly modify tumour growth. NO inhibits proliferation of tumour cells (Lepoivre et al, 1989; Maragos et al, 1993; Jenkins et al, 1995) and tumour-infiltrating lymphocytes (Lejeune et al, 1994). It also induces apoptosis in malignant cells and reduces formation of metastases (Xie et al, 1995*a,b*). In vivo, synthesis of NO at low levels stimulates angiogenesis leading to accelerated tumour growth (Jenkins et al, 1995). Treatment with 5,6-MeXAA induces NO synthesis in tumour-bearing and in healthy mice (Thomsen et al, 1990, 1991). NO synthesis, measured by an increase in its oxidation products in plasma, in response to 5,6-MeXAA and chemical analogues of flavone acetic acid correlates with the delay in tumour growth caused by these agents in a colon 38 tumour model in mice (Thomsen et al, 1991). Mouse peritoneal macrophages activated by *Bacillus Calmette-Guerin* synthesized NO in response to 5,6-MeXAA in vitro (Thomsen et al, 1990). The aim of the present study was to investigate the profile of 5,6-MeXAA-induced NO synthesis in tumour and other tissues and the relationship between NO synthesis and the anti-tumour action of 5,6-MeXAA in immunocompetent mice bearing subcutaneous colon 38 tumours.

### MATERIALS AND METHODS

### Materials

5,6-MeXAA was synthesized at the Cancer Research Laboratory, University of Auckland, Auckland, New Zealand (Rewcastle et al,

**Table 1** NOS activity in tissues before (control) and 12 h after treatment with 5,6-MeXAA (27.5 mg kg$^{-1}$ i.p.) in tumour-bearing B6D2F1 mice

| Tissue | NOS (pmol min$^{-1}$ mg$^{-1}$ protein; mean ± s.e.m.[a]) | | |
|---|---|---|---|
| | Total | $Ca^{2+}$-dependent | $Ca^{2+}$-independent |
| Tumour | | | |
| Control | 1.5 ± 0.4 | 0.2 ± 0.1 | 1.3 ± 0.4 |
| 12 h | 8.6 ± 2.1**[b] | 0.8 ± 0.3 | 7.8 ± 1.9** |
| Spleen | | | |
| Control | 0.7 ± 0.2 | 0.4 ± 0.1 | 0.3 ± 0.2 |
| 12 h | 4.9 ± 0.7*** | 0.8 ± 0.2 | 4.1 ± 0.6*** |
| Thymus | | | |
| Control | 1.9 ± 1.2 | 1.0 ± 1.0 | 1.0 ± 0.4 |
| 12 h | 5.3 ± 1.0* | 0.8 ± 0.7 | 4.5 ± 0.5** |
| Intestine | | | |
| Control | 0.5 ± 0.2 | 0.2 ± 0.1 | 0.3 ± 0.1 |
| 12 h | 2.3 ± 0.3*** | 0.5 ± 0.2 | 1.8 ± 0.3*** |
| Lung | | | |
| Control | 3.1 ± 0.5 | 2.6 ± 0.4 | 0.5 ± 0.1 |
| 12 h | 3.4 ± 0.6 | 2.7 ± 0.2 | 0.7 ± 0.3 |
| Kidney | | | |
| Control | 3.3 ± 0.3 | 2.4 ± 0.2 | 0.9 ± 0.1 |
| 12 h | 3.4 ± 0.3 | 2.6 ± 0.3 | 0.8 ± 0.1 |
| Skeletal muscle | | | |
| Control | 7.3 ± 1.2 | 3.8 ± 0.7 | 3.5 ± 0.7 |
| 12 h | 7.1 ± 1.3 | 3.9 ± 1.6 | 2.9 ± 0.7 |
| Liver | | | |
| Control | 3.6 ± 1.0 | ND[c] | ND |
| 12 h | 4.0 ± 2.2 | ND | ND |
| Heart | | | |
| Control | 0.2 ± 0.1 | ND | ND |
| 12 h | 0.6 ± 0.4 | ND | ND |

[a]5 or 6 mice per group; [b]differences between corresponding values before and 12 h after 5,6-MeXAA are denoted by *$P$ < 0.05, **$P$ < 0.01 and ***$P$ < 0.001; [c]ND, not determined.

1991), and was a generous gift from Professor BC Baguley. $N$-iminoethyl-L-ornithine (L-NIO) and $N^G$-monomethyl-L-arginine (L-NMMA) were synthesized by the Department of Medicinal Chemistry at the Wellcome Research Laboratories, Beckenham, UK. L-[U$^{14}$C]Arginine was purchased from Amersham (Buckinghamshire, UK), cell culture reagents from Gibco (Paisley, UK), reagents for immunohistochemistry from Dako (Bucks, UK) and other chemicals were from Sigma, Boehringer Mannheim or BDH unless otherwise indicated.

### Animal procedures

B6D2F1 mice were purchased from Harlan, Oxon, UK and housed at a constant temperature and humidity with regular 12-h cycles of light and darkness, sterile bedding, water and food. All experiments were carried out under institutional ethical guidelines. In experiments with tumour-bearing animals, colon 38 tumours were implanted subcutaneously and allowed to grow to a diameter of 5–10 mm before use. 5,6-MeXAA was dissolved in 5% bicarbonate and administered as a single intraperitoneal (i.p.) or subcutaneous (s.c.) injection. Lipopolysaccharide (LPS) (4 mg kg$^{-1}$, TCA extract of *Salmonella typhimurium*, Sigma) and killed *Corynebacterium parvum* organisms (100 mg kg$^{-1}$, Wellcome) were dissolved in sterile phosphate-buffered saline (PBS) and injected i.p. After the time interval indicated, mice were anaesthetized with halothane and blood was collected into heparinized tubes. Animals were killed by cervical dislocation, resident peritoneal macrophages were harvested in sterile PBS (5 ml per mouse) and tissues collected and snap-frozen in liquid nitrogen.

When tumour growth was studied, tumours were measured for length and width by calipers every second day after treatment with 5,6-MeXAA. The tumour volume was estimated using the formula: tumour volume = 0.52 × length × width$^2$.

### Measurement of NOS activity in tissues

Frozen tissues were thawed in ice-cold 20 mM Hepes buffer (pH 7.2) containing 200 mM sucrose, 0.1 mM EDTA, 5 mM dithiothreitol, 10 μg ml$^{-1}$ leupeptin, 10 μg ml$^{-1}$ soyabean trypsin inhibitor and 1 μg ml$^{-1}$ pepstatin A and homogenized (with an Ystral homogenizer) in ice. The homogenates were centrifuged at 10 000 ***g*** at 2°C for 30 min. Supernatants were treated with equal volume of cation-exchange resin (Dowex-50W, sodium form) to remove endogenous arginine. NOS activity in the supernatants (cytosol + microsomes) was measured by the conversion of L-[U-$^{14}$C]arginine to [U-$^{14}$C]citrulline at 37°C for 10 min in 20 mM Hepes buffer (pH 7.2) containing 10 μM tetrahydrobiopterin, 2.5 mM dithiothreitol, 400 U ml$^{-1}$ calmodulin, 250 μM calcium chloride, 0.5 mg ml$^{-1}$ bovine serum albumin, 125 μM NADPH, 10 μM arginine, 100 μM citrulline, 60 mM valine (to inhibit arginase) and 0.33 μCi ml$^{-1}$ L-[U-$^{14}$C]arginine, as described previously (Salter et al, 1991). The total NOS activity was determined from the difference between the [U-$^{14}$C]citrulline generated in control samples and samples containing 1 mM L-NMMA; the activity of the calcium-dependent NOS activity was determined from the difference between control samples and samples containing 1 mM EGTA, and the activity of calcium-independent NOS was determined from the difference between samples containing 1 mM EGTA and those with 1 mM L-NMMA. In liver supernatants, addition of EGTA resulted in an anomalous apparent increase in NOS activity, so that it was not possible to determine the calcium-dependent and calcium-independent NOS activities separately. Protein content of the tissue supernatants was measured by Coomassie brilliant blue assay (Bio-Rad) using bovine plasma albumin as a standard.

### Measurement of NOS activity in peritoneal macrophages

The peritoneal cells were spun down and resuspended in Dulbecco's modification of Eagle medium containing 100 μM L-arginine, 10% heat-inactivated fetal calf serum, penicillin (100 U ml$^{-1}$) and streptomycin (100 U ml$^{-1}$). The macrophages were allowed to adhere on tissue culture plates for 2 h. Thereafter, the plates were washed twice with sterile PBS to remove non-adherent cells. The adherent cells were harvested with a plastic scraper into homogenization buffer and snap-frozen in liquid nitrogen. NOS activity in the cytosol was measured as described above, except that the cells were disrupted by sonication instead of mechanical homogenization. Macrophages from six mice were pooled to obtain each value.

### Immunocytochemistry

Sections (5 μm thick) were cut from frozen tissue, air dried for 30 min and fixed for a further 30 min in acetone. Endogenous peroxidase was visualized with 0.05% DAB (Diaminobenzidene)

© Cancer Research Campaign 1998

**Figure 1** Time courses of the stimulatory action of 5,6-MeXAA (27.5 mg kg$^{-1}$ i.p.) on plasma NOx concentrations (○) and on NOS activity in thymus (●), spleen (▼) and in tumour (■ in the inset) in tumour-bearing B6D2F1 mice. Mean ± s.e.m. ($n$ = 6)

and non-specific binding sites were blocked with 20% normal rabbit or swine sera for 15 min. A polyclonal primary antibody against murine iNOS raised in rabbit (Anti-macNOS, Transduction Laboratories, Lexington, KY, USA) was used at a 1:50 dilution to detect iNOS. A rat antibody against murine CD31 (Clone 390, Pharmingen, c/o Cambridge Biosciences, Cambridge, UK) was applied at a 1:100 dilution for the detection of endothelial cells (PECAM-1). For the demonstration of murine macrophages, MOMA-2 (Serotec, Oxford, UK) was used at a dilution of 1:10. The tissue sections were incubated with the optimally diluted primary antibody for 30 min at room temperature. Slides were then washed in Tris-buffered saline, and covered with biotinylated rabbit anti-rat IgG or swine anti-rabbit IgG. After further rinsing, slides were incubated with horseradish peroxidase-conjugated streptavidin. The slides were then developed using aminoethylcarbizole and counterstained with haematoxylin.

### Plasma nitrite + nitrate (NOx) assays

Plasma samples were diluted with distilled water, and proteins were precipitated with zinc sulphate. Nitrate was reduced to nitrite with acid-washed cadmium (Davison et al, 1978), and thereafter nitrite concentrations were measured using a microplate assay based on the Griess reaction (Green et al, 1982).

### TNF and other parameters measured in plasma

TNF concentrations in the plasma were measured by enzyme-linked immunosorbent assay (ELISA), as described previously (Deakin et al, 1995). Plasma urea concentrations were assayed by using a colorimetric kit for urea nitrogen (Sigma). Creatinine, glutamate dehydrogenase (GLDH) and alanine transferase (ALT) levels were assayed in plasma samples using standard clinical chemistry methods using a Roche Cobas Biocentrifugal Analyser.

### Statistics

Results are expressed as means ± standard error of the mean (s.e.m.). When indicated, statistical significance was calculated by analysis of variance supported by Bonferroni adjusted significance levels. Differences were considered significant when $P < 0.05$.

### RESULTS

### Induction of NOS activity in tissues

NOS activity in tissue extracts was measured 12 h after 5,6-MeXAA (27.5 mg kg$^{-1}$ i.p.) administration, which represents the peak level of plasma NOx in tumour-bearing animals (see below). Calcium-independent NOS activity was increased in the tumour, spleen, thymus and small intestine, whereas NOS activity in the lung, liver, kidney, heart and skeletal muscle was unchanged (Table 1). The time-courses of NOS activity in the spleen, thymus and tumour were measured. NOS activities in the thymus and spleen peaked at 12 h after treatment with 5,6-MeXAA and decreased thereafter, mimicking the time-course of increases in plasma NOx (Figure 1). NOS activity in the tumour, however, continued to increase up to 3 days after 5,6-MeXAA treatment (Figure 1). The peak NOS activity in the tumours (25 pmol min$^{-1}$ mg$^{-1}$ protein) was higher than that of any of the other tissues studied.

© Cancer Research Campaign 1998

**Figure 2** Immunohistochemical studies of tumour tissue. Sections of tumour tissue before treatment with 5,6-MeXAA, labelled with antibody against iNOS (**A**) and (**C**), endothelial cells CD31 (**B**) and macrophage marker MOMA-2 (**D**), showing iNOS localized to endothelial cells within the tumour and in some macrophages in the tumour capsule. Sections of tumour tissue taken 3 days after treatment, immunolabelled with antibody against iNOS (**E**) and macrophage marker MOMA-2 (**F**), showing significant necrosis of the tumour tissue and macrophages within and around the tumour expressing iNOS (bar = 40 μm)

Calcium-independent NOS activity in peritoneal macrophages from tumour-bearing mice after treatment with 5,6-MeXAA (27.5 mg $kg^{-1}$) was increased from 2 pmol $min^{-1}$ $mg^{-1}$ protein to 16 (12 h after treatment) and 31 (3 days after treatment). The increases were lower than those caused by two other immunostimulatory compounds, LPS (71 pmol $min^{-1}$ $mg^{-1}$ protein 8 h after treatment) and *Corynebacterium parvum* (92 pmol $min^{-1}$ $mg^{-1}$ protein 7 days after treatment) in macrophages from control, non-tumour-bearing animals.

### Immunohistochemical localization of iNOS in the tumour

Before treatment with 5,6-MeXAA, iNOS activity was present in endothelial cells of the tumour as defined by the CD31 antibody (Figure 2A,B). Some macrophages around the capsule, as defined by the macrophage marker MOMA-2, also stained positively for iNOS (Figure 2C and D). Twelve hours, 1, 3 and 7 days after treatment with 5,6-MeXAA, significant necrosis in the tumour and

© Cancer Research Campaign 1998

**Figure 3** The time–response curves of the stimulatory effects of 5,6-MeXAA (**A**) and LPS (**B**) on plasma NOx concentrations in normal B6D2F1 mice. Mean ± s.e.m. ($n$ = 6). (**A**) ◆, 5,6-MeXAA 40 mg kg$^{-1}$ s.c.; ■, 5,6-MeXAA 40 mg kg$^{-1}$ i.p.; ●, 5,6-MeXAA 27.5 mg kg$^{-1}$ i.p.; ○, 5,6-MeXAA 27.5 mg kg$^{-1}$ i.p. + L-NIO (30 mg kg$^{-1}$ before and 100 mg kg$^{-1}$ 5 h after 5,6-MeXAA). (**B**) ●, LPS (4 mg kg$^{-1}$) i.p.; ○ LPS (4 mg kg$^{-1}$) i.p. + L-NIO (30 mg kg$^{-1}$ before and 100 mg kg$^{-1}$ 5 h after LPS)

**Figure 4** 5,6-MeXAA-induced accumulation of NOx in plasma in tumour-bearing and control B6D2F1 mice. Mean ± s.e.m. ($n$ = 6). ○, 5,6-MeXAA 27.5 mg kg$^{-1}$ i.p. in tumour-bearing mice; ●, 5,6-MeXAA 27.5 mg kg$^{-1}$ i.p. in control mice; ■, 5,6-MeXAA 40 mg kg$^{-1}$ i.p. in control mice infiltrated macrophages were noted, with a marked reduction in endothelial cells. Macrophages within and around the tumour expressed iNOS at 12 h, 1, 3 and 7 days after 5,6-MeXAA treatment (example at 3 days, Figure 2E and F). Expression of iNOS by tumour cells was not observed at any time.

### Increase in plasma NOx concentrations

5,6-MeXAA increased plasma NOx in control, non-tumour-bearing mice after i.p. and s.c. administration, reaching peak concentrations 8–12 h after dosing (Figure 3A). Plasma NOx concentrations returned to their initial levels within 24 h after 5,6-MeXAA injections. The increase in plasma NOx was inhibited by L-NIO, an inhibitor of NOS, in a dose-dependent manner. L-NIO completely blocked increases in plasma NOx when it was given at 30 mg kg$^{-1}$ before and 100 mg kg$^{-1}$ 5 h after 5,6-MeXAA (27.5 mg kg$^{-1}$ i.p.) (Figure 3A). A lower dose of L-NIO (30 mg kg$^{-1}$ before and 30 mg kg$^{-1}$ 5 h after 5,6-MeXAA) reduced plasma NOx concentrations measured 12 h after 5,6-MeXAA administration by 83%. The corresponding inhibitory action was 68% when L-NIO was given in two 10 mg kg$^{-1}$ doses. The time course and the increase in plasma NOx concentrations induced by 5,6-MeXAA was similar to those caused by LPS (4 mg kg$^{-1}$) (Figure 3B).

The maximal tolerated doses of 5,6-MeXAA in tumour-bearing B6D2F1 mice were lower (27.5 mg kg$^{-1}$) than in non-tumour-bearing controls (40 mg kg$^{-1}$). The effect of 5,6-MeXAA on plasma NOx was greater in tumour-bearing than in control mice (Figure 4).

As measured in control mice, the increase in plasma NOx was preceded by a moderate increase in plasma TNF concentrations and haematocrit (Figure 5) and accompanied by a rise in plasma urea levels and the appearance of the liver enzymes GLDH and ALT in the plasma (Table 2).

### Effects of L-NIO on the tumoricidal action of 5,6-MeXAA

To understand the role of NO in the tumoricidal action of 5,6-MeXAA, mice with subcutaneous colon 38 tumours were treated with L-NIO. A dosing schedule of 60 mg kg$^{-1}$ twice a day s.c. was used. This was based on the inhibitory effects of similar dosing regimens of L-NIO on the increase in plasma NOx induced by 5,6-MeXAA and LPS (see results described above and Figure 3). The first dose was injected 2 h after 5,6-MeXAA and this was repeated at 12-h intervals for 7 days thereafter. Treatment with this regimen of L-NIO did not alter the tumoricidal activity of 5,6-MeXAA (Figure 6).

### DISCUSSION

Treatment with 5,6-MeXAA increased NO production, as evidenced by increased NOS activity in the tumour and some other tissues and by enhanced NOx concentrations in plasma. Calcium-independent but not calcium-dependent NOS activity was increased, indicating induction of iNOS (Knowles et al, 1994).

© Cancer Research Campaign 1998

**Figure 5** Time courses of the effects of 5,6-MeXAA (40 mg $kg^{-1}$ i.p.) on TNF concentrations in plasma (●), haematocrit (○) and the accumulation of NOx in plasma (■) in normal B6D2F1 mice. Mean ± s.e.m. ($n$ = 6)

**Table 2** Effects of 5,6-MeXAA (40 mg $kg^{-1}$ i.p.) on plasma NOx concentrations and indicators of liver and kidney function in normal B6D2F1 mice

| Parameter | Before 5,6-MeXAA | 12 h after 5,6-MeXAA | 24 h after 5,6-MeXAA |
|---|---|---|---|
| NOx (µM) | 73 ± 13[a] | 866 ± 80***[b] | 133 ± 19 |
| Urea (mM) | 7.5 ± 0.7 | 14.9 ± 1.3*** | 10.0 ± 0.7 |
| Creatinine (µM) | 39.4 ± 1.7 | 36.0 ± 2.1 | 33.0 ± 2.0 |
| ALT (U $ml^{-1}$) | 30.6 ± 1.7 | 73.6 ± 10.2** | 43.3 ± 4.1 |
| GLDH (U $ml^{-1}$) | 6.6 ± 0.7 | 16.2 ± 1.4*** | 12.3 ± 2.3 * |

[a]Mean ± s.e.m. $n$ = 5 or 6 mice per group; [b]Differences from corresponding value before 5,6-MeXAA are denoted by *$P$ < 0.05, **$P$ < 0.01 and ***$P$ < 0.001.

The increase in calcium-independent NOS activity was evident in immunologically active organs (i.e. thymus, spleen, small intestine) and in peritoneal macrophages as well as in the tumour. Immunostaining showed that the predominant cell type in the tumour that stained with the iNOS antibody was the infiltrating macrophages, but not the tumour cell itself. This provides further evidence that at least in this model it is the immune cells that generate NO after treatment with 5,6-MeXAA.

The time-course as well as the tissue selectivity of the induction of iNOS activity by 5,6-MeXAA was quite distinct from those of other immunostimulatory agents known to induce iNOS. 5,6-MeXAA induced NOS activity in thymus, spleen, small intestine and in the tumour but not in other tissues such as lung, liver and heart and thus differs from the action of LPS, muramyl dipeptide and heat-inactivated *Corynebacterium parvum* (Palacios et al, 1992; Cunha et al, 1994; Rees et al, 1995). Induction of iNOS was present a few hours after treatment with 5,6-MeXAA, LPS or myramyl dipeptide, whereas administration of heat-inactivated *Corynebacterium parvum* induced NO synthase with a time course of several days (Palacios et al, 1992; Cunha et al, 1994; Rees et al, 1995). The iNOS immunoreactivity in the tumour after treatment with 5,6-MeXAA was associated with macrophages. However, iNOS activity in peritoneal macrophages induced by 5,6-MeXAA was low compared with the activity in macrophages after treatment with LPS or *Corynebacterium parvum*. These data suggest that the role of NO as an effector mechanism in activated macrophages and other target cells is different depending on the immunostimulant used.

In vitro, 5,6-MeXAA is able to induce NO synthesis in mouse peritoneal macrophages activated by Bacillus Calmette-Guerin but not in resident or thioglycollate-elicited macrophages (Thomsen et al, 1990). These data suggest that 5,6-MeXAA alone is not a sufficient stimulus to induce iNOS in murine macrophages but that other activating or priming agents are required. The increase in plasma NOx concentrations was higher in tumour-bearing than non-tumour-bearing animals after equivalent doses of 5,6-MeXAA. The same phenomenon has been reported after treatment with flavone-8-acetic acid, a compound related to 5,6-MeXAA (Thomsen et al, 1991). This could be due to NO production in the tumour cells as well as due to tumour-induced activation of the immune cells (Scheiber et al, 1995) leading to higher response to immunostimulatory agents. In earlier experiments (Thomsen et al, 1991), the higher increase in plasma NOx after 5,6-MeXAA in tumour-bearing animals was present, although the tumours were cut out just before the treatment with 5,6-MeXAA. In the present study, iNOS was induced in tumour-associated macrophages but not in the tumour cells. These data suggest that NO production in the tumour cells does not explain the higher response in plasma NOx after 5,6-MeXAA in tumour-bearing mice than in control mice. Immunoreactive tissues such as spleen, thymus and lymph nodes may be a source of increased plasma NOx. However, in separate studies, we have been unable to detect increased iNOS activity in spleen from tumour-bearing compared with non tumour-bearing mice (LL Thomsen, unpublished observation). It could therefore be hypothesized that tumour antigens and/or products provide the priming stimuli required for 5,6-MeXAA to elicit iNOS induction in the tumour-associated macrophages and immunologically active organs.

The response to treatment with 5,6-MeXAA was characterized by high concentrations of plasma NOx that peaked 8–12 h after injection of the compound and was associated with biochemical

© Cancer Research Campaign 1998 

**Figure 6** Effects of L-NIO on the anti-tumour action of 5,6-MeXAA. Animals with subcutaneously growing colon 38 tumours were treated with 5,6-MeXAA on day 0 (27.5 mg kg$^{-1}$ i.p.) (■), 5,6-MeXAA (27.5 mg kg$^{-1}$ i.p.) followed by L-NIO (60 mg kg$^{-1}$ s.c. twice a day for 7 days from day 0) (▼) or with vehicle only (○). Mean ± s.e.m. ($n$ = 8)

alterations (ie. greater than twofold increases from basal levels of plasma urea, and liver enzymes ALT and GLDH) and clinical signs such as lethargy and dyspnoea, found in septic shock-like syndrome. These biochemical and clinical signs, plasma NOx concentrations, as well as NOS activity in thymus and spleen, returned towards their initial levels in 24 h, whereas the NOS activity in the tumour macrophages continued to increase and reached its peak at 3 days after treatment with 5,6-MeXAA. Tumour-associated macrophages have a pleiotropic function in the regulation of tumour growth (Mantovani et al, 1992). These cells produce growth factors and stimulate angiogenesis, thus augmenting tumour growth. In contrast, tumour-associated macrophages can be activated to inhibit tumour growth and destroy neoplastic cells (Mantovani et al, 1992). The potential tumoricidal mechanisms of activated macrophages in vitro include increased synthesis of NO (Hibbs et al, 1987; Cox et al, 1992; Lorsbach et al, 1993). However, recent studies with transfected tumour cell lines constitutively expressing iNOS at relatively low levels, have demonstrated that NO augments tumour growth in vivo probably because of enhanced angiogenesis (Jenkins et al, 1995). To understand the role of enhanced NO synthesis in tumour-associated macrophages after treatment with 5,6-MeXAA, the animals were treated with a combination of 5,6-MeXAA and L-NIO, an inhibitor of NOS enzymes. L-NIO at the regime given did not alter significantly the tumoricidal activity of 5,6-MeXAA, suggesting that NO may not be a major mediator in the anti-tumour action of 5,6-MeXAA. A lack of effect of NOS inhibitors on the tumour necrosis caused by some derivatives of flavone acetic acid and TNF has been reported by Veszelovszky et al (1993). However, the haemodynamic effects of L-NIO acting on eNOS in the vasculature may contribute to the response and further studies with selective inhibitors of iNOS or with iNOS knock-out mice are needed to establish definitively what the role of NO is in the tumoricidal action of 5,6-MeXAA.

Increased concentrations of plasma NOx have been measured during cancer immunotherapy with interleukin 2 (Hibbs et al, 1992; Ochoa et al, 1992; Thomsen et al, 1992; Miles et al, 1994) and increased NO production has been associated with the side-effects of this cytokine (Kilbourn et al, 1994; Miles et al, 1994). Thus, combining a NOS inhibitor with cancer immunotherapy with agents such as interleukin 2 and 5,6-MeXAA, which themselves increase NOS activity, provides a theoretical means to diminish the side-effects and/or increase maximal tolerated doses to achieve a better anti-tumour action.

In conclusion, an experimental anti-tumour agent 5,6-MeXAA, which has been selected for clinical evaluation, was shown to induce NO synthesis in immunologically active tissues and in tumour-associated macrophages. Further experiments are needed to understand the role, if any, of enhanced NOS activity in the anti-tumour action or side-effects of 5,6-MeXAA.

### ACKNOWLEDGEMENTS

The authors wish to acknowledge Professor BC Baguley and Dr DC Jenkins for their expert advice during the study, Mr N Davies for his experienced technical assistance with the animal studies and Dr David Smith for help in analyses of basic clinical chemistry. Dr Eeva Moilanen received a grant from the Academy of Finland.

### REFERENCES

Ching L-M, Joseph WR and Baguley BC (1992) Stimulation of macrophage tumoricidal activity by 5,6-dimethyl-xanthone-4-acetic acid, a potent analogue of the antitumor agent flavone-8-acetic acid. *Biochem Pharmacol* **44**: 192–195

Ching L-M, Joseph WR, Crosier KE and Baguley BC (1994*a*) Induction of tumor necrosis factor-α messenger RNA in human and murine cells by the flavone acetic acid analogue 5,6-dimethylxanthenone-4-acetic acid (NSC 640488). *Cancer Res* **54**: 870–872

Ching L-M, Joseph WR, Zhuang L and Baguley BC (1994*b*) Interaction between the antitumor agent 5,6-dimethylxanthenone-4-acetic acid in the induction of tumor necrosis factor and haemorrhagic necrosis of colon 38 tumors. *Cancer Chemother Pharmacol* **35**: 153–160

Cox GW, Melillo G, Chattopadhyay U, Mullet D, Fertel RH and Varesio L (1992) Tumor necrosis factor-α-dependent production of reactive nitrogen intermediates mediates IFNγ plus IL-2-induced murine macrophage tumoricidal activity. *J Immunol* **149**: 3290–3296

Cunha FQ, Assreuy J, Moss DW, Rees D, Leal LMC, Moncada S, Carrier M, O'Donnell CA and Liew FY (1994) Differential induction of nitric oxide synthase in various organs of the mouse during endotoxaemia: role of TNF-α and IL-1-β. *Immunology* **81**: 211–215

Davison W and Woof C (1978) Comparison of different forms of cadmium as reducing agents for the batch determination of nitrate. *Analyst* **103**: 403–406

Deakin AM, Payne AN, Whittle BJR and Moncada S (1995) The modulation of IL-6 and TNF-α release by nitric oxide following stimulation of J774 cells with LPS and IFN-γ. *Cytokine* **7**: 408–416

Futami H, Eader L, Back TT, Gruys E, Young HA, Wiltrout RH and Baguley BC (1992) Cytokine induction and therapeutic synergy with IL-2 against murine renal cancer by xanthenone-4-acetic acid derivatives. *J Immunother* **12**: 247–255

Green LC, Wagner DA, Glogowski J, Skipper PL, Wishnok JS and Tannenbaum SR (1982) Analysis of nitrate, nitrite and [$^{15}$N]nitrate in biological fluids. *Anal Biochem* **126**: 131–138

Hibbs J, Taintor RR and Vavrin Z (1987) Macrophage cytotoxicity: role for L-arginine deiminase and imino nitrogen oxidation to nitrite. *Science* **235**: 473–476

Hibbs JB, Westenfelder C, Taintor R, Vavrin Z, Kablitz C, Baranowski RL, Ward JH, Menlove RL, McMurry MP, Kushner JP and Samlowski WE (1992) Evidence of cytokine-inducible nitric oxide synthesis from L-arginine in patients receiving interleukin-2 therapy. *J Clin Invest* **89**: 867–877

Jenkins CD, Charles IG, Thomsen LL, Moss DW, Holmes LS, Baylis SA, Rhodes P, Westmore K, Emson PC and Moncada S (1995) Roles of nitric oxide in tumor growth. *Proc Natl Acad Sci USA* **92**: 4392–4396

Kilbourn RG, Owen-Schaub LB, Cromeens DM, Gross SS, Flaherty MJ, Santee SM, Alak AM and Griffith OW (1994) N-methyl-L-arginine, an inhibitor of nitric oxide formation, reverses IL-2-mediated hypotension in dogs. *J Appl Physiol* **76**: 1130–1137

Knowles RG and Moncada S (1994) Nitric oxide synthases in mammals. *Biochem J* **298**: 249–258

Lejeune P, Lagadec P, Onier N, Pinard D, Ohshima H and Jeannin JF (1994) Nitric oxide involvement in tumor-induced immunosuppression. *J Immunol* **152**: 5077–5083

© Cancer Research Campaign 1998

Lepoivre M, Boudbid H and Petit J-F (1989) Antiproliferative activity of γ-interferon combined with lipopolysaccharide on murine adenocarcinoma: Dependence on an L-arginine metabolism with production of nitrite and citrulline. *Cancer Res* **49**: 1970–1979

Lorsbach RB, Murphy WJ, Lowenstein CJ, Synder SH and Russell SW (1993) Expression of the nitric oxide synthase gene in mouse macrophages activated for tumor cell killing. *J Biol Chem* **268**: 1908–1913

Maragos CM, Wang JM, Hrabie JA, Oppenheim JJ and Keefer LK (1993) Nitric oxide/nucleophile complexes inhibit the in vitro proliferation of A375 melanoma cells via nitric oxide release. *Cancer Res* **53**: 564–568

Mantovani A, Bottazzi B, Colotta F, Sozzani S and Ruco L (1992) The origin and function of tumor-associated macrophages. *Immunol Today* **13**: 265–270

Miles D, Thomsen L, Balkwill F, Thavasu P and Moncada S (1994) Association between biosynthesis of nitric oxide and changes in immunological and vascular parameters in patients treated with interleukin-2. *Eur J Clin Invest* **24**: 287–290

Ochoa JB, Curti B, Peitzman AB, Simmons RL, Billiar TR, Hoffmann R, Rault R, Longo DL, Urba WJ and Ochoa AC (1992) Increased circulating nitrogen oxides after human tumor immunotherapy: Correlation with toxic hemodynamic changes. *J Natl Cancer Inst* **84**: 864–867

Palacios M, Knowles RG and Moncada S (1992) Enhancers of nonspecific immunity induce nitric oxide synthase: induction does not correlate with toxicity or adjuvancy. *Eur J Immunol* **22**: 2303–2307

Perera P-Y, Barber SA, Ching L-M and Vogel SN (1994) Activation of LPS-inducible genes by the antitumor agent 5,6-dimethylxanthenone-4-acetic acid in primary murine macrophages. *J Immunol* **153**: 4684–4693

Rees DD, Cunha FQ, Assreuy J, Herman AG and Moncada S (1995) Sequential induction of nitric oxide synthase by *Corynebacterium parvum* in different organs of the mouse. *Br J Pharmacol* **114**: 689–693

Rewcastle GW, Atwell GJ, Zhuang L, Baguley BC and Denny WA (1991) Potential antitumor agents. 61. Structure-activation relationships for in vivo colon 38 activity among disubstituted 9-oxo-9H-xanthene-4-acetic acids. *J Med Chem* **34**: 217–222

Salter M, Knowles RG and Moncada S (1991) Widespread tissue distribution, species distribution and changes in activity of $Ca^{2+}$-dependent and $Ca^{2+}$-independent nitric oxide synthases. *FEBS Lett* **291**: 145–149

Scheiber H and Rowley DA (1995) The immunology of solid tumors. In *Samter's Immunologic Diseases*. Frank MM, Austen KF, Claman HN and Unanue ER (eds), pp. 607–622. Little, Brown: Boston

Thomsen LL, Ching L-M and Baguley BC (1990) Evidence for the production of nitric oxide by activated macrophages treated with the antitumor agents flavone-8-acetic acid and xanthenone-4-acetic acid. *Cancer Res* **50**: 6966–6970

Thomsen LL, Ching L-M, Zhuang L, Gavin JB and Baguley BC (1991) Tumor-dependent increased plasma nitrate concentrations as an indicator of the antitumor effect of flavone-8-acetic acid and analogues in mice. *Cancer Res* **51**: 77–81

Thomsen LL, Baguley BC, Rustin GJ and O'Reilly SM (1992) Flavone acetic acid (FAA) with recombinant interleukin-2 (IL-2) in advanced malignant melanoma. II: induction of nitric oxide production. *Br J Cancer* **66**: 723–727

Veszelovszky E, Thomsen LL, Zhuang L and Baguley BC (1993) Flavone acetic acid and 5,6-dimethylxanthenone-4-acetic acid: Relationship between plasma nitrate elevation and the induction of tumor necrosis. *Eur J Cancer* **29A**: 404–408

Xie K, Huang S, Dong Z, Juang S-H, Gutman M, Xie Q-W, Nathan C and Fidler IJ (1995*a*) Transfection with the inducible nitric oxide synthase gene suppresses tumorigenity and abrogates metastasis by K-1735 murine melanoma cells. *J Exp Med* **181**: 1333–1343

Xie K, Huang S, Dong Z, Gutman M and Fidler IJ (1995*b*) Direct correlation between expression of endogenous nitric oxide synthase and regression of M5076 reticulum cell sarcoma hepatic metastases in mice treated with liposomes containing lipopeptide CGP 31362. *Cancer Res* **55**: 3123–3131

Zwi LJ, Baguley BC, Gavin JB and Wilson WR (1989) Blood flow failure as a major determinant in the antitumor action of flavone acetic acid. *J Natl Cancer Inst* **81**: 1005–1013

© Cancer Research Campaign 1998